# A Genome-Wide Association Study Identifying Novel Genetic Markers of Response to Treatment with Interleukin-23 Inhibitors in Psoriasis

**DOI:** 10.3390/genes16101195

**Published:** 2025-10-13

**Authors:** Sophia Zachari, Kalliopi Liadaki, Angeliki Planaki, Efterpi Zafiriou, Olga Kouvarou, Kalliopi Gerogianni, Themistoklis Giannoulis, Zissis Mamuris, Dimitrios P. Bogdanos, Nicholas K. Moschonas, Theologia Sarafidou

**Affiliations:** 1 Department of Biochemistry and Biotechnology, University of Thessaly, Viopolis, 41500 Larissa, Greece; sofizach2@uth.gr (S.Z.); kliad@bio.uth.gr (K.L.); aplanaki@uth.gr (A.P.); zmamur@uth.gr (Z.M.); 2 Department of Dermatology, Faculty of Medicine, School of Health Sciences, University of Thessaly, Viopolis, 41500 Larissa, Greece; zafevi@med.uth.gr (E.Z.); kouvarouolga@gmail.com (O.K.); kalgerog@yahoo.com (K.G.); 3 Department of Animal Science, University of Thessaly, Gaiopolis, 41334 Larissa, Greece; themisgia@gmail.com; 4 Department of Rheumatology and Clinical Immunology, Faculty of Medicine, School of Health Sciences, University of Thessaly, Viopolis, 41500 Larissa, Greece; bogdanos@med.uth.gr; 5 School of Medicine, University of Patras, 26500 Patras, Greece; 6 Foundation for Research and Technology Hellas, Institute of Chemical Engineering Sciences, 26504 Patras, Greece

**Keywords:** Guselkumab, Risankizumab, Interleukin-23 inhibitors, psoriasis, genome-wide association study

## Abstract

**Background/Objectives**: The advent of biologics targeting key inflammatory pathways has significantly advanced psoriasis treatment. Among them, the Interleukin-23 inhibitors Guselkumab and Risankizumab have demonstrated high efficacy and rapid clinical response in both clinical trials and real-world studies. However, up to 30% of patients fail to respond. This study aimed to identify pharmacogenetic markers associated with treatment response using a genome-wide association study (GWAS) and protein network-based approach. **Methods**: Fifty-three patients of Greek origin with moderate-to-severe plaque psoriasis were treated with Guselkumab or Risankizumab. Based on Psoriasis Area and Severity Index (PASI) improvement at 3 and 6 months, patients were categorized as responders or non-responders. Approximately 730,000 single-nucleotide polymorphisms (SNPs) were genotyped. After filtering, a GWAS was performed to identify variants associated with treatment response. Additionally, protein–protein interaction (PPI) network analysis was applied to the two Interleukin-23 subunits and SNPs within or near genes encoding Interleukin-23-interacting proteins to test for their association. **Results**: The GWAS identified two novel variants, rs73641950 and rs6627462, significantly associated with treatment response, with both surpassing the genome-wide significance threshold after Bonferroni correction. The PPI-based approach revealed rs13086445, located downstream of the Interleukin-12 subunit alpha (IL12A) gene, as another associated variant. All three SNPs lie in genomic regions with potential regulatory roles. **Conclusions**: This study identifies three novel genetic variants associated with response to Interleukin-23 inhibitors in psoriasis. These findings provide promising pharmacogenetic markers which, upon validation in larger, independent cohorts, will enable the translation of a patient’s genotype into a response phenotype, thereby guiding clinical decisions and improving drug effectiveness.

## 1. Introduction

Psoriasis affects more than 125 million people globally and has no cure. The condition causes skin plaques from overactive immune cells and increases the risk of diabetes, cardiovascular diseases, and psoriatic arthritis, as well as impacting mental health due to reduced quality of life and stigmatization [1]. The introduction of biologics has revolutionized the treatment of psoriasis. The first biologic therapy, which targeted tumor necrosis factor alpha (TNF-a), was approved by the Food and Drug Administration (FDA) in 2002. Today, in addition to TNF-a inhibitors, there are biologic agents that specifically target cytokines such as interleukin-17A (IL-17A) and its receptor (IL17RA), interleukin-17F (IL-17F), interleukin-23 (IL-23), interleukins 12/23 (IL-12/23), and the interleukin-36 receptor (IL-36R), with the latter being targeted in generalized pustular psoriasis [2].

The pro-inflammatory cytokine IL-23, consisting of two subunits, IL23A (p19) and IL12B (p40), is released by dendritic cells or macrophages and plays a number of important roles in innate and adaptive immunity [3]. IL-23 has been characterized as a key factor in psoriasis pathogenesis by genetic and immunological studies [4]. To date, three IL-23 inhibitors have been approved for adults with moderate-to-severe plaque psoriasis: Guselkumab (Tremfya^®^) in 2017, Tildrakizumab (Ilumetri^®^) in 2018, and Risankizumab (Skyrizi^®^) in 2019. Guselkumab and Risankizumab have also been approved for the treatment of psoriatic arthritis, Crohn’s disease, and ulcerative colitis. All IL-23 inhibitors are administered subcutaneously, although they differ in their dosing intervals. These agents specifically target the p19 subunit of IL-23, thereby disrupting the IL-23/IL-17 axis without inhibiting the inteleukin-12/T helper type 1 (IL-12/Th1) pathway, which has been suggested to not be essential or even to have potential protective effects [5]. They exert their action by blocking the binding of IL-23 to its receptor (IL-23R), thereby preventing the expansion of T helper 17 (Th17) cells and the production of pro-inflammatory cytokines, such as IL-17 and IL-22 [6]. One of the key advantages of IL-23 p19 inhibitors is their high effectiveness. Alongside IL-17 inhibitors, they demonstrate the fastest clinical response among systemic therapies [7] and have been shown to induce clinical remission in both biologic-naïve and biologic-experienced patients [8]. Additionally, these agents, particular Guselkumab and Risankizumab, are associated with high drug survival rates [9].

Pooled clinical trials of IL-23 inhibitors have demonstrated disease improvement in over 70% of patients [10,11,12,13,14,15]. A recent review summarizing data from 74 real-world studies reports that the effectiveness and safety profile of these inhibitors are comparable to those observed in clinical trials [16]. However, despite their proven utility, IL-23 inhibitors do not lead to clinical improvement in a significant proportion of patients. This lack of response can severely affect patients’ quality of life and contributes to an unnecessary economic burden. This heterogeneity in treatment response is considered a multifactorial trait, influenced by both genetic and clinical factors, such as lifestyle, disease duration, and comorbidities [17]. A number of pharmacogenetic markers associated with response to various biologic treatments in psoriasis have been identified through association studies. These markers are classified into different levels of evidence according to the Pharmacogenetics and Pharmacogenomics Knowledge Base, PharmGKB (www.pharmgkb.org/), (accessed on 28 April 2025). However, none of these markers are currently used in clinical practice, highlighting the need for robust, clinically applicable biomarkers to support personalized medicine. In this context, research on pharmacogenetic markers specific to IL-23 inhibitors remains limited. To our knowledge, only one recent study has examined this topic, in an investigation of the association of 21 pre-selected SNPs with treatment response to Guselkumab or Risankizumab in a cohort of 18 patients from Spain [18]. The authors reported associations between three single-nucleotide polymorphisms (SNPs) and treatment response, but only after 12 months of therapy and when patients were classified as responders based on absolute Psoriasis Area and Severity Index (PASI) scores [19] (PASI < 1).

The primary objective of this study was to conduct the first genome-wide association study (GWAS) of treatment response to the IL-23 inhibitors Guselkumab and Risankizumab in a Greek cohort of 53 patients with psoriasis. Secondary objectives included (a) analyzing associations between polymorphisms in candidate genes based on the protein–protein interaction (PPI) network of the IL-23 subunits, and (b) replicating previously reported associations with IL-23 treatment response [18].

## 2. Materials and Methods

We followed the guidelines of the Equator Network (https://www.equator-network.org/), (accessed on 2 September 2025) for a pharmacogenetic study described in [20] and included the STROPS (STrengthening the Reporting Of Pharmacogenetic Studies) criteria compliance checklist (Table S1).

### 2.1. Patients and Treatment Response

This prospective cohort pharmacogenetic study included a total of 55 Greek patients (out of 63 potentially eligible patients) treated for psoriasis with Guselkumab or Risankizumab as monotherapy at the Dermatology Department of the General University Hospital of Larissa, Greece. Patients were recruited during the period between September 2021 and June 2024. The inclusion criteria for the patients to be involved in the study were as follows: (i) diagnosis of moderate-to-severe plaque psoriasis with a PASI score ≥ 5; (ii) age > 18 years; (iii) continuous treatment for at least 6 months after the initial starting dose; and (iv) systematic follow-up during the period between October 2021 and January 2025. Patients with other irrelevant concomitant autoimmune diseases or pregnant women were excluded from the study. The dosing of Guselkumab and Risankizumab was 100 mg and 150 mg, respectively, administered subcutaneously at week 0, at week 4, and then every 12 weeks. At week 0, the baseline PASI scores, age of disease onset, body weight and height, the presence of comorbidities and the respective pharmacotherapy, psoriatic arthritis (PsA) and nail psoriasis, and whether patients were biologic-naïve or biologic-experienced were recorded. Follow-up was performed in the context of scheduled check-ups at weeks 4, 12, 16, and 24, during which the PASI was assessed. Adherence to treatment was assessed via self-report and/or direct observation. The baseline PASI score threshold of 5 is consistent with many real-world studies, which better reflect clinical practice and include a broader range of patient populations [16] compared to clinical trials of IL-23p19 inhibitors [10,11,12,13,14,15]. In our cohort, the baseline PASI scores ranged from 5 to 12.5 in 35 patients, ranged from 12.5 to 20 in 11 patients, and were greater than 20 in 7 patients. Out of the total 53 patients, 9 had been previously treated with apremilast, and this cohort subset was included in our previous pharmacogenetic analysis of treatment response to apremilast [21].

Patients were categorized as responders or non-responders at 3 and 6 months, based on improvement in their PASI score. Responders were patients that achieved PASI 75 or greater after 12 or 24 weeks of treatment, whereas non-responders showed less than 50% improvement in their PASI score. In addition, since the treatment standard for psoriasis tends to be the achievement of a PASI 90 response [22], patients were categorized as responders if they achieved PASI 90 or greater, and as non-responders if they showed less than PASI 75 improvement. The PASI measurements at baseline, 3 months, and 6 months were assessed within a two-week time window. A comparison of patients’ characteristics between responders and non-responders was performed using Fisher’s statistical significance exact test [23] for categorical variables such as gender, comorbidities, and biologic-naïve status. For all other characteristics, the nonparametric Kruskal–Wallis test for comparing more than two independent samples [23] was applied, as the data deviated from the normal distribution. All analyses were conducted in Rstudio (version 4.1 2), a statistical programming language [24].

This study was approved by the Research Ethics Committee of the University General Hospital of Larissa, University of Thessaly (ECA #19/14-11-2019 and ECA 11/02/08-02-2022), with the informed consent of the patients. All patients provided a written consent to participate in the study, and the study was conducted in accordance with the World Medical Association (WMA) Declaration of Helsinki—Ethical Principles for Medical Research Involving Human Participants (www.wma.net/policies-post/wma-declaration-of-helsinki/), (accessed on 3 July 2025).

### 2.2. Genomic DNA Isolation and Genotyping

Peripheral blood collected from patients in the presence of anticoagulant was stored in 2 mL aliquots at −20 °C until further use. Genomic DNA was extracted using the PureLink Genomic DNA kit (Thermo Fisher Scientific, Waltham, Massachusetts, United States) kit from 200 μL peripheral blood. The Quawell spectrophotometer and agarose gel electrophoresis were used to evaluate the concentration, purity, and integrity of the DNA preparation. Genomic DNAs were stored at −20 °C. Each DNA sample was adjusted to a concentration of 50 ng/μL and 4 μL volumes were sent in dry ice to the Human Genomics Facility (HuGe-F) (www.hugef.nl/), (accessed on 30 April 2025) of Erasmus Medical Centre, The Netherlands, for genotyping with the high-throughput Illumina Infinium Global Screening Array Multiple Disease v3.0. This array contains approximately 730,000 variants and is enriched in variants associated with diseases. The patients’ DNA samples were included in two 96-well plates with other human DNA samples that were not relevant to this study.

### 2.3. Quality Control, Filtering, and Association Analysis

The genotyping data, obtained from .ped and .map files, were subjected to quality control using a filtering pipeline in the following order: (i) missingness filtering removed SNPs and individuals with >1% missing data across all individuals and >1% missing genotype data, respectively; (ii) extreme heterozygosity filtering removed individuals with values greater than the mean ± 5 SD; (iii) SNPs with a minor allele frequency (MAF) of ≤5% were excluded; (iv) variants in high linkage disequilibrium (LD) (threshold set at r^2^ > 0.8) were pruned based on pairwise correlation, with the window for the calculation set to 50 SNPs and the window shifting 5 SNPs forward; (v) individuals with more than a fourth-degree relation were removed through identity-by-descent (IBD) analysis with a threshold set at the kinship coefficient PI_HAT value > 0.0625. All the above analyses were performed using the whole-genome association analysis toolset PLINK 1.9 [25]. Power calculations were performed in Rstudio [24] using the genpwr.calc function. With the given sample size, assuming that 20% of the patients are non-responders (cases)—a mean estimate based on the literature [10,11,12,13,14,15,16]—and with alpha (α) = 0.05, the statistical power primarily depends on the odds ratio (OR) and the minor allele frequency (MAF). Our a priori calculations indicated that for a MAF of 20%, a high OR would be required to achieve reasonable power. For example, for a MAF of 20% and an OR of 2, the power was only 23%; with a MAF of 30% and an OR of 3, the power increased to 55.2%. Therefore, we designed the study with the expectation of detecting variants with a relatively high MAF and large OR.

### 2.4. Databases

dbSNP [26], (www.ncbi.nlm.nih.gov/snp/) was used to identify the chromosomal location of variants and their genomic context (accessed on 10 July 2025). The Ensembl database [27] (https://www.ensembl.org/index.html, accessed on 10 July 2025) was exploited to find the coordinates of genes using the human genome assembly GRCh37 in order to achieve compatibility with the genotyping data derived from HuGe-F (www.hugef.nl/), (accessed on 30 April 2025) the minor allele frequency (MAF) of variants in European populations and variants in high linkage disequilibrium. PICKLE, the protein–protein interaction (PPI) meta-database for the direct protein–protein interactome of human and mouse proteomes (release 3.3), [28,29], (www.pickle.gr) was used to extract PPI data for human IL-23 subunits by applying the “first neighbors” setup and the “cross-checking” filtering method (accessed on 5 May 2025). HaploReg v4.2 (https://pubs.broadinstitute.org/mammals/haploreg/haploreg.php), a tool that explores annotations of the noncoding genome at variants on haplotype blocks [30], was searched to explore the effect of SNPs on regulatory motifs and SNPs in high linkage disequilibrium (accessed on 30 July 2025). The Cytoscape software [31] for visualizing and integrating complex networks, version 3.8.2, was used for the reconstruction of the PPI network. The expression quantitative trait locus (eQTL) effect of the variants was evaluated through the Genotype-Tissue Expression (GTEx) portal [32], (www.gtexportal.org), (accessed on 10 June 2025). The UniProt knowledgebase (www.uniprot.org/uniprotkb/), (accessed on 20 June 2025), the central hub for the collection of functional information on proteins, with accurate and rich annotation [33], was used to retrieve the approved human gene and protein names and symbols.

## 3. Results

### 3.1. Patient Characteristics and Treatment Outcomes

**Figure 1:**
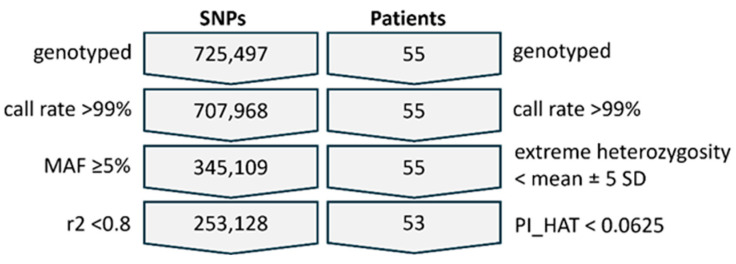
Quality control and filtering steps for SNPs and patients. Fifty-three patients remained suitable for analysis, and 253,128 high-quality SNPs were finally retained for the genome-wide association analysis.

Out of the 55 patients with psoriasis initially enrolled in the study, 2 were excluded after quality control of the genotyping data due to cryptic relatedness (PI_HAT value > 0.0625). Overall, a total of 53 patients (32 males and 21 females) were included in the association analysis (Figure 1).

Table 1 summarizes the demographic and clinical characteristics of the patients. The mean age of psoriasis onset was 36.7 years (±16.4), and the mean age at initiation of treatment with IL-23 inhibitors was 56.1 years (±13.3). A high percentage of the patients (74%) had been previously treated with other biological drugs, given that IL-23 inhibitors were approved more recently than anti-TNF and anti-IL17 therapies and the mean duration of disease prior to the initiation of anti-IL23 treatment in this cohort was approximately 20 years.

At baseline, the mean PASI was 10.4 (±9.1), the mean weight was 89.2 kg (±20.4), and the mean BMI was 30.9 (±7.3). Approximately 48% of the patients were classified as obese (BMI > 30), and 28% were classified as overweight (BMI > 25). Among the patients, the prevalence of nail psoriasis and psoriatic arthritis was 56% and 36%, respectively. Comorbidities (which are common in patients with long-lasting psoriasis), including cardiovascular disease, diabetes mellitus, depression, and hypothyroidism, were reported in 65% of the patients.

Regarding treatment, 21 of the 53 patients were administered Guselkumab, while 32 received Risankizumab. Treatment response was evaluated at two time points, 3 months and 6 months post treatment, with a threshold of 75% or 90% improvement in the PASI. The 3-month evaluation showed that 43 out of 50 patients and 38 out of 45 patients were responders based on PASI 75 and PASI 90, respectively. At 6 months, 47 out of 53 patients achieved a PASI 75 response, and 46 out of 52 achieved a PASI 90 response. Notably, 18 out of 53 patients were categorized as super-responders (PASI 100) after 3 months of treatment, and this number increased to 33 out of 53 patients at 6 months.

To examine whether any patient characteristics are associated with the efficacy of IL-23 treatment, we compared baseline data between responder and non-responder groups. This subgroup analysis found no significant differences in gender, age of treatment initiation, biologic-naïve status, baseline PASI, weight, BMI, or the presence of comorbidities (Table S2). The only statistically significant difference was in the age of disease onset, which was higher in non-responders. This finding suggests that late-onset psoriasis may be associated with a reduced response to treatment, possibly due to age-related factors.

### 3.2. GWAS of Treatment Response

After quality control and filtering of the genotyping data, 253.128 SNPs with a minor allele frequency (MAF) of > 5% were included in the GWAS of response to treatment with IL-23 inhibitors (Figure 1). The analysis, based on the allelic test, identified two SNPs that remained significant after Bonferroni correction, i.e., rs73641950 (T/C) and rs6627462 (C/T) (Table 2). The results of the association analyses for all the SNPs are given in Supplementary Tables S3–S6.

For both SNPs, the frequency of the rare allele (C and T, respectively) was significantly higher in the non-responder group, showing that it constitutes a risk allele. Notably, rs73641950 was associated with treatment response at both time points (3 and 6 months), regardless of the PASI threshold (PASI 75 or PASI 90). In contrast, rs6627462 was only associated with the 3-month response. However, the raw *p*-values for this SNP at 6 months were 1.6 × 10^−6^ for PASI 75 and 2 × 10^−6^ for PASI 90, which could reach statistical significance with a larger sample size.

Overall, the MAFs of rs73641950 and rs6627462 in the entire cohort were 7.5% and 21.6%, respectively, which are similar to the frequencies reported for European populations (8% and 22%) in the Ensembl database. Regarding their chromosomal location, rs73641950 is located within an intron of the *NFIB* gene, according to dbSNP (NCBI), and rs6627462 is an intergenic variant on the X chromosome, located less than 13 kb from the *CNGA2* gene, which encodes the alpha subunit of a cyclic nucleotide-gated olfactory channel.

### 3.3. Association Analysis for Candidate Genes

**Figure 2:**
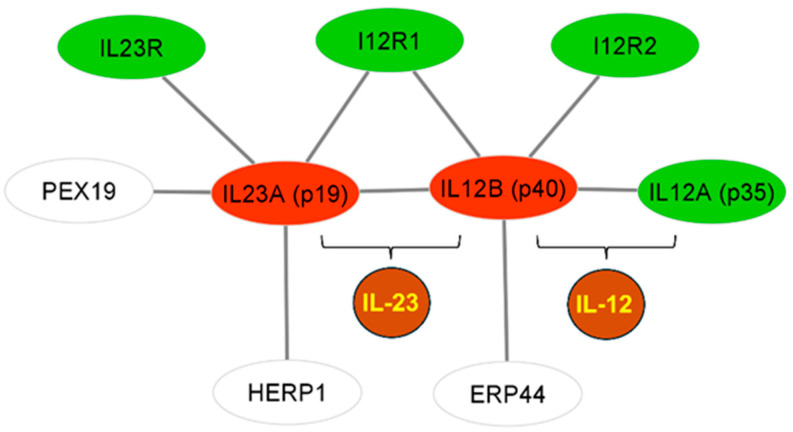
Reconstruction of the IL-23 PPI network. IL-23, consisting of the IL23A (p19) and IL12B (p40) subunits (illustrated in red ovals), directly interacts with seven proteins, including IL23R, I12R1, I12R2, and the IL12A (p35) subunit of IL-12 (all illustrated in green ovals), and with three non-cytokine proteins: PEX19, HERP1 and ERP44 (illustrated in white ovals). IL12B (p40) is the common subunit shared by IL-23 and IL-12 (both illustrated in brown disks).

SNPs within or near the two genes, *IL23A* and *IL12B*, encoding for the IL-23 protein subunits IL23A (p19) and IL12B (p40), respectively, and genes whose products interact with the IL-23 heterodimer are, in principle, primary candidates for association with the drug response. To carry out a systematic SNP–gene association, we first reconstructed the PPI network of IL-23 with its first neighbors. IL23A (p19) and IL12B (p40) directly interact with seven proteins (Figure 2). Namely, IL23R and I12R1 form the two subunits of the IL23 receptor, which is essential in IL-23 signal transduction [34]; I12R1 associates with I12R2 to form a high-affinity receptor for the heterodimer IL-12 which consists of IL12B (p40) and IL12A (p35) subunits [35]. PEX19 participates in peroxisomal biogenesis, neutral lipid storage, and lipid droplet dynamics [36]; recently, pathogenic mutations in *PEX19* have been identified in patients with the autosomal recessive Zellweger syndrome [37]. Finally, HERP1 and ERP44 have functional roles in the endoplasmic reticulum; HERP1 contributes to the endoplasmic reticulum-associated degradation of misfolded proteins [38], and ERP44 contributes to oxidative protein folding [39].

We selected all SNPs located withinthe nine genes of the protein network, as well as SNPs within a physical distance of approximately 1 Mb upstream and downstream of the genomic region of all genes. This cis-distance threshold is commonly used in eQTL calculations [40]. We then tested for allelic association with IL-23 response. In total, 1304 SNPs (Table S7) within or near each gene were included in the analysis, with 63 of them found with nominal *p* values < 0.05 (Table S8). After Bonferroni correction, one SNP, rs13086445 (T/G), located 950 kb downstream of *IL12A*, remained statistically significant with a *p* value ranging from 0.0026 to 0.0282, depending on the time point (3 or 6 months) and the PASI threshold for response (PASI75 or PASI90) (Table 3).

### 3.4. Replication Analysis

A crucial step in validating SNPs detected in association analyses and establishing robust genetic markers for treatment response prediction is the independent replication of previous findings. To date, only one pharmacogenetic study on treatment response to IL-23 inhibitors has been published [18], which identified three SNPs in the *TLR2* (rs11938228), *TLR5* (rs5744174), and *ANKRD55* (rs96844) genes as being associated with treatment response after 12 months, with raw *p*-values of 0.0418, 0.0143, and 0.038, respectively. We investigated the raw data for these SNPs and their potential association. All three SNPs are indeed included in our genotyping dataset, but they do not show an association with treatment response at either 3 or 6 months (raw *p*-values > 0.2).

## 4. Discussion

Using a genome-wide association approach, we conducted the first pharmacogenetic analysis of response to the IL-23 inhibitors Guselkumab and Risankizumab. The analysis identified a genome-wide significant association between the variants rs73641950 and rs6627462, and treatment response in patients with moderate-to-severe psoriasis. The minor alleles of these variants appear to act as risk alleles for non-response, as their frequencies were markedly increased in the non-responder group. The odds ratios (ORs) for these associations were notably high compared to those typically observed in genome-wide association studies (GWAS), suggesting strong effects; however, the low allele counts likely contributed substantially to these elevated values. The minor allele frequencies (MAFs) were approximately 7% for rs73641950 and approximately 20% for rs6627462, which is located on the X chromosome. Despite this, the associations remained statistically significant after Bonferroni correction with 95% confidence intervals (CIs) that did not include 1. Given the high ORs, standard power estimation may not be appropriate; therefore, we used the lower bounds of the 95% CIs for power calculation, resulting in an estimated power between 50.3 and 59.4% for rs73641950 and between 81.6 and 82.3% for rs6627462.

Variant rs73641950 is located in an intronic region of the *NFIB* gene, which encodes a transcription factor involved in organ development and tumorigenesis [41]. Although *NFIB* has not been linked to psoriasis or other autoimmune diseases, a recent study in chicken cells showed that its overexpression increased inflammatory cytokine levels via activation of the NF-κB pathway, mediated by upregulation of RIP2 [42]. As no functional data are currently available for rs73641950, its potential impact remains speculative. However, based on its location, it may influence *NFIB* expression through regulatory mechanisms. According to HaploReg v4.2, rs73641950 lies within binding motifs for Smad, Smad3, and ATF3 [43,44]. Notably, it is in high linkage disequilibrium (D’ = 0.94, r^2^ = 0.81) with rs16931840, located 11 kb upstream, which overlaps a DNase I hypersensitive site found in several cell types, including skin-derived foreskin melanocytes. These findings suggest that rs73641950 may have a regulatory role affecting *NFIB* or nearby gene expression.

Variant rs6627462 is intergenic, with the nearest gene, *CNGA2* (13 kb upstream), encoding a subunit of the olfactory channel that is active in sensory neurons [45]. According to GTEx, this variant is not a cis-eQTL in psoriasis-relevant tissues, so its functional role in the drug’s mechanism cannot be inferred. However, although many GWAS variants lack functional characterization, they may still serve as valuable genetic markers of drug response once validated.

In addition to the GWAS, a network-based approach was used to identify genes encoding direct protein interactors of IL-23 subunits as potential candidates for treatment response. This analysis identified one variant, rs13086445, located 950 kb downstream of the *IL12A* gene (dbSNP). To date, this variant has not been linked to *IL12A* function or expression (GTEx), so its functional role in relation to this cytokine remains unclear. However, it lies within overlapping binding motifs for Crx1, Nrf-2, Pou1f1, and Pou5f1 (HaploReg v4.2), suggesting that the alternative allele may affect transcription factor binding.

Finally, we conducted a replication analysis for SNPs in the *TLR2*, *TLR5*, and *ANKRD55* genes, which were previously associated with treatment response to IL-23 inhibitors in a pre-selected SNP association study of a cohort of 18 patients with moderate-to-severe psoriasis from Spain [18]. However, these findings were not replicated in the current study, which may be due to differences in the time point of patient response evaluation (12 months vs. 3 or 6 months in the current study) and the method used to classify responders and non-responders (absolute PASI vs. percentage of PASI improvement).

Our analysis identified three promising pharmacogenetic markers associated with treatment response to Guselkumab and Risankizumab, two of which reached genome-wide significance, underscoring the potential for personalized therapy in psoriasis. However, the small sample size (n = 53) limits the applicability of these findings. Validation in larger, independent, and gender-stratified cohorts is essential, particularly for variant rs6627462 on chromosome X, which may be influenced by X inactivation and dosage compensation mechanisms. These variants represent preliminary markers that require clinical validation across diverse populations, supported by meta-analyses. Upon confirmation, development of a regulatory-approved genotyping assay and its integration into clinical guidelines could enable pharmacogenetic testing to reduce trial-and-error prescribing and enhance cost-effective, personalized psoriasis management.

## 5. Conclusions

This study, the first genome-wide association study (GWAS) to investigate the relationship between genetic markers and positive response to IL-23 inhibitor treatment in psoriasis, identified two novel variants, rs73641950 and rs6627462, that reached genome-wide significance. Additionally, a protein network-based approach identified another novel variant, rs13086445, located downstream of the *IL12A* gene, as being associated with treatment response. These variants require further investigation, including validation in independent larger cohorts and functional characterization to investigate their causality.

## Figures and Tables

**Table 1:** Clinical and demographic characteristics of the patients.

Characteristics	Patients
Gender (males/females)	32/21
Age of disease onset (mean ± SD) years	36.7 (±16.4)
Age of treatment onset (mean ± SD) years	56.1 (±13.3)
Biologic-naïve patients * (%)	26%
Baseline PASI (mean ± SD)	10.4 (±9.1)
Baseline weight (kg) (mean ± SD)	89.2 (±20.4)
Baseline Body Mass Index (BMI) (mean ± SD)	30.9 (±7.3)
Comorbidities (%)	65%

* This information was available for 38 patients. Note: SD: standard deviation.

**Table 2:** Statistically significant SNPs associated with the response to Guselkumab and Risankizumab.

	rs73641950; Chr. 9: 14.499.258 bp Intronic, *NFIB* Gene					rs6627462; Chr. X: 151.758.565 bp Intergenic				
	F_R	F_NR	*p* Adj	OR	95% CI	F_R	F_NR	*p* Adj	OR	95% CI
3 m.: R (n = 43) > PASI 75 NR (n = 7) < PASI 50	0.012	0.429	0.0036	63.75	7–597	0.100	0.889	0.0103	72	7.6–679
3 m.: R (n = 38) > PASI 90 NR (n = 7) < PASI75	0.013	0.429	0.0244	56.18	6–528	0.096	0.889	0.0208	75	7.7–731
6 m.: R (n = 47) > PASI 75 NR (n = 6) < PASI 50	0.021	0.500	0.0009	46.08	7.6–278					
6 m.: R (n = 46) > PASI 90 NR (n = 6) < PASI 75	0.022	0.500	0.0013	45.05	7.4–273					

Note: 3 m.: 3 months; 6 m.: 6 months; R: responders; NR: non-responders; F_R: frequency of the alternative allele in responders; F_NR: frequency of the alternative allele in non-responders; *p* adj.: *p* adjusted; OR: odds ratio; CI: confidence interval.

**Table 3:** Allele frequencies of variant rs13086445 in responder (R) and non-responder (NR) groups at 3 and 6 months of treatment, and at PASI75 and PASI90 response thresholds.

	F_R	F_NR	Raw *p* Value	*p* Adj	OR	95% CI
3 m.: R (n = 43) > PASI 75 NR (n = 7) < PASI 50	0.023	0.357	5.6 × 10^−6^	0.0073	23.3	3.9–138
3 m.: R (n = 38) > PASI 90 NR (n = 7) < PASI75	0.026	0.357	2.2 × 10^−5^	0.0282	20.6	3.5–122
6 m.: R (n = 47) > PASI 75 NR (n = 6) < PASI 50	0.032	0.417	2.0 × 10^−6^	0.0026	21.7	4.3–110
6 m.: R (n = 46) > PASI 90 NR (n = 6) < PASI 75	0.033	0.417	2.7 × 10^−6^	0.0035	21.2	4.2–108

Note: 3 m.: 3 months; 6 m.: 6 months; R: responders; NR: non-responders; F_R: frequency of the alternative allele in responders; F_NR: frequency of the alternative allele in non-responders; *p* adj.: *p* adjusted; OR: odds ratio; CI: confidence interval.

## Data Availability

The original contributions presented in the study are included in the article’s Supplementary Material; further inquiries can be directed to the corresponding authors.

## Supplementary Materials

The following supporting information can be downloaded at https://www.mdpi.com/article/10.3390/genes16101195/s1. Table S1: Strengthening the Reporting of Pharmacogenetic Studies (STROPS) guideline compliance checklist; Table S2: Comparison of patients’ characteristics between responder and non-responder groups; Table S3: Association analysis for 3-month treatment response, setting PASI 75 as the threshold; Table S4: Association analysis for 3-month treatment response, setting PASI 90 as the threshold; Table S5: Association analysis for 6-month treatment response, setting PASI 75 as the threshold; Table S6: Association analysis for 6-month treatment response, setting PASI 90 as the threshold. Table S7: SNPs located within or near each gene encoding the nodes of the IL-23 PPI network; Table S8: SNPs within or near IL-23 and its first neighbors showing nominal *p*-values < 0.05.

## Abbreviations

The following abbreviations are used in this manuscript:

TNF	Tumor necrosis factor
IL	Interleukin
PASI	Psoriasis Area and Severity Index
PPI	Protein–protein interaction
GWAS	Genome-wide association study
eQTL	Expression quantitative trait locus
SNP	Single-nucleotide polymorphism
