# Supplementary material for: A Genome-Wide Association Study Identifying Novel Genetic Markers of Response to Treatment with Interleukin-23 Inhibitors in Psoriasis
Source: Genes (Basel). 2025 Oct 13;16(10):1195. doi: 10.3390/genes16101195 (PMC12564705; doi:10.3390/genes16101195)
Supplement: Supplementary file 1 [file genes-16-01195-s001.zip › Zachari et al_genes-3835404_Table S1.pdf]

Table S1. STrengthening the Reporting Of Pharmacogenetic Studies (STROPS) guideline compliance checklist.

Category	#	Criteria	Section, Table, Figure
**Abstract**			
Abstract	1	Provide in the abstract an informative and balanced summary of what was done and what was found.	Manuscript abstract
**Introduction**			
Background/ rationale	2	Explain the scientific background and rationale for the investigation being reported.	Introduction section (3rd paragraph)
	3	Provide reasons for choosing the genes and SNPs genotyped.	This is a GWAS study and not a candidate SNP/gene approach.
Objectives	4	State specific objectives, including any prespecified hypotheses.	Last paragraph of the Introduction section.
	5	State if the study is the first report of a pharmacogenetic association, a replication effort, or both.	Last paragraph of the Introduction section.
**Methods**			
Study design	6	Present key elements of study design early in the paper.	Section 2.1
Setting	7	Describe the setting, locations, and relevant dates, including periods of recruitment, follow-up, and data collection.	Section 2.1
Participants	8	Give the eligibility criteria and the sources and methods of selection of participants. For a cohort study, describe methods of	Section 2.1

Category	#	Criteria	Section, Table, Figure
		follow-up. For a case-control study, state whether true controls or population controls were used. Give the rationale for the choice of cases and controls.	
	9	Report the drug and regime participants were exposed to and the length of exposure.	Section 2.1
	10	For a matched case-control study, give matching criteria and the number of controls per case.	Not applicable. We did not analyze matched case-control studies
	11	Give information on the criteria and methods for selection of subsets of participants from a larger study, when relevant.	Not applicable. We did not selected participants from a larger study.
	12	If other publications report results for the same patient cohort or a subset of the patient cohort, provide information on this patient cohort overlap and references to the relevant publications.	Section 2.1 and reference #21
	13	Report disease/clinical indication of patients using a standardized ontology when possible.	MeSH terms used: Plaque Psoriasis; Psoriatic Arthritis, Nail Psoriasis
Variables	14	Clearly define all outcomes, potential confounders, and effect modifiers. Give diagnostic criteria, if applicable.	Section 2.1; last paragraph of section 3.1
	15	Provide justification for choice of outcomes.	Section 2.1

Category	#	Criteria	Section, Table, Figure
	16	Clearly define genetic exposures (genetic variants) using a widely used nomenclature system.	Section 2.2
	17	Report the rs number of each genotyped SNP.	Tables 2 and 3
	18	Clearly state how haplotypes or star alleles were defined.	Not applicable. We did not use haplotypes or star alleles.
	19	If referring to the minor, major, wild-type, mutant, reference, risk or effect allele of a variant, state which allele this is and for which given population/cohort.	Sections 3.2 and 3.3
Data sources/ measurement	20	For each variable of interest, give sources of data and details of methods of assessment (measurement). Describe comparability of assessment methods if there is more than one group.	Section 2.1 and reference #19
	21	Describe laboratory methods, including source and storage of DNA, genotyping methods and platforms (including the allele calling algorithm used, and its version), error rates, and call rates. State the laboratory/center where genotyping was done. Describe comparability of laboratory methods if there is more than one group. Specify whether genotypes were assigned using all of the data from the study	Sections 2.2 and 2.3

Category	#	Criteria	Section, Table, Figure
		simultaneously or in smaller batches.	
	22	Describe genotype quality control methods and findings.	Section 2.3 & Figure 1
	23	For quantitative outcome variables, specify if any investigation of potential bias resulting from pharmacotherapy was undertaken. If relevant, describe the nature and magnitude of the potential bias, and explain what approach was used to deal with this.	Stratification of the patients based on pharmacotherapy that could influence treatment response was not performed due to small sample size but, as far as it is known from clinical trials results, the pharmacokinetic properties of IL-23 inhibitors were not affected by the specific pharmacotherapy.
	24	Report how adherence to treatment was assessed, and report the results of the assessment.	Section 2.1
Study size	25	Explain how the study size was arrived at, or provide details of the a priori power to detect effect sizes of varying degrees.	Section 2.3
Quantitative variables	26	Explain how quantitative variables (confounders and effect modifiers) were handled in the analyses. If applicable, describe which groupings were chosen, and why.	Not applicable. Continuous variables have not been grouped into categories.
Statistical methods	27	Address the following:	
	(a)	Describe methods used to control for confounding.	Not applicable. Adjustment was not performed for disease onset between responders and non-responders

Category	#	Criteria	Section, Table, Figure
	(b)	Describe any methods used to examine subgroups and interactions.	Not applicable. We did not have subgroups.
	(c)	Explain how missing data were addressed.	Table S2
	(d)	Cohort study—If applicable, explain how loss to follow-up was addressed.	Section 2.1. Individuals lost during follow-up were excluded from the study.
	(e)	Case-control study—If applicable, explain how matching of cases and controls was addressed.	Not applicable. This is not a matched case-control study.
	(f)	Describe any sensitivity analyses.	Not applicable, no sensitivity analysis was performed.
	28	State whether Hardy–Weinberg equilibrium was considered, and if so, how.	Not applicable. Hardy–Weinberg equilibrium was not considered since all the participants are patients.
	29	Describe any methods used for inferring genotypes or haplotypes.	Not applicable. Genotypes or haplotypes have not been inferred.
	30	Describe any methods used to assess or address population stratification.	Not applicable. Population stratification was not addressed.
	31	Describe any methods used to assess and correct for relatedness among subjects. Report results of assessments for relatedness.	Section 2.3 and Figure 1.
	32	Describe any methods used to address multiple comparisons or to control risk of false positive results due to (a) multiple genetic variants, (b) multiple outcomes, and (c) multiple	(a) Section 3.2<br>(b): not applicable. Multiple outcomes have not been examined.

Category	#	Criteria	Section, Table, Figure
		assumptions regarding mode of inheritance. Allelic test	(c): Not applicable. Allelic association tests have been only performed.
	33	Describe any methods used to adjust for extent of adherence in the analyses.	Not applicable. We consider that all the patients that were finally included in the study adhered to the treatment which was checked as described in Section 2.1.
**Results**			
Participants	34	Report the numbers of individuals at each stage of the study—e.g., numbers potentially eligible, examined for eligibility, confirmed eligible, included in the study, completing follow-up, and analyzed.	Section 2.1
SNPs	35	Report any SNPs that were excluded from analysis, and provide reasons for these exclusions.	Figure 1
Descriptive data	36	Give characteristics of study participants (e.g., demographic, clinical, social, ethnicity) and information on potential confounders.	Table 1 and Section 2.1
	37	Cohort study—Summarize follow-up time, e.g., average and/or total amount.	Section 2.1
	38	Where HWE tests have been undertaken, highlight SNPs that deviate from HWE.	Not applicable. Hardy–Weinberg equilibrium was not considered since all the participants are patients.

Category	#	Criteria	Section, Table, Figure
	39	Where population stratification is assessed, report the results.	Not applicable. Population stratification was not addressed.
Outcome data	40a	For a cohort study, report all outcomes (phenotypes) investigated for each genotype category over time.	Table 2 and Table 3.
	40b	For a case-control study, report numbers in each genotype category for all outcomes investigated.	Not applicable. This is not a case-control study.
	40c	For a cross-sectional study, report all outcomes (phenotypes) investigated for each genotype category.	Not applicable. This is not a cross-sectional study.
	41	If a study includes more than one ethnic group, provide the summary data specified in (40) per ethnic group.	Not applicable. The study includes only one ethnic group.
Main results	42	Give unadjusted estimates, and if applicable, confounder-adjusted estimates and their precision (e.g., 95% confidence intervals). Make clear which confounders were adjusted for and why they were included.	Not applicable. Adjustment was not performed for disease onset between responders and non-responders
	43	Report category boundaries when continuous variables were categorized.	Not applicable. Continuous variables were not categorized.
Other analyses	44	Report other analyses done—e.g., analyses of subgroups and interactions, and sensitivity analyses.	Not applicable. We did not have subgroups.
	45	If numerous genetic exposures (genetic	Tables S3-S8

Category	#	Criteria	Section, Table, Figure
		variants) were examined, summarize results from all analyses undertaken.	
	46	If detailed results are available elsewhere, i.e., in supplementary materials, state how they can be accessed.	Tables S3-S8 are cited in the manuscript (sections 3.2 and 3.3).
**Discussion**			
Key results	47	Summarize key results with reference to study objectives.	Discussion section (1st paragraph).
Limitations	48	Discuss limitations of the study, taking into account sources of potential bias or imprecision. Discuss both direction and magnitude of any potential bias.	Discussion section (last paragraph).
Interpretation	49	Give a cautious overall interpretation of results considering objectives, limitations, multiplicity of analyses, results from similar studies, and other relevant evidence.	Discussion section
Generalizability	50	Discuss the generalizability (external validity) of the study results.	Discussion section
**Other information**			
Study registration	51	State whether the study has been registered. If the study has been registered, provide details of the registry.	Not applicable. The study has not been registered in any platform.
Ethical approval	52	Report whether ethical approval was obtained for the collection of genetic data.	Section 2.1

Category	#	Criteria	Section, Table, Figure
Funding	53	Give the source of funding and the role of the funders for the present study, and if applicable, for the original study on which the present article is based.	Not applicable. This research received no external funding.
Databases	54	State whether databases for the analyzed data are or will become publicly available, and if so, how they can be accessed.	Files of analyzed data are linked to the online version of the paper.

HWE, Hardy–Weinberg equilibrium; rs, reference SNP cluster ID; SNP, single nucleotide polymorphism.
